# Senescent immune cells, inflammaging, and bone loss: therapeutic potential and translational challenges of senolytics in osteoporosis

**DOI:** 10.3389/fendo.2026.1917096

**Published:** 2026-08-24

**Authors:** Minshun Zhu, Jie Zhang, Houshan Fang, Kuo Cai, Qing Wu, Changping Gao, Yueling Wang, Jiaping Chen

**Affiliations:** Department of Rehabilitation, Lu’an Hospital of Traditional Chinese Medicine, Lu’an, China

**Keywords:** bone remodeling, immunosenescence, inflammaging, osteoimmunology, osteoporosis, senescence-associated secretory phenotype, senolytics

## Abstract

Osteoporosis is a common age-related skeletal disorder characterized by low bone mass, deterioration of bone microarchitecture, and increased susceptibility to fragility fractures. Although antiresorptive and anabolic therapies have substantially improved fracture prevention, current treatments do not fully address the aging-related biological processes that disrupt skeletal homeostasis. Increasing evidence from osteoimmunology, immunosenescence, and cellular senescence research suggests that inflammaging contributes to age-related bone loss by reshaping the bone marrow immune microenvironment. Immune cells with senescence-associated features may promote bone remodeling imbalance through excessive production of senescence-associated secretory phenotype factors, including interleukin-1β, interleukin-6, tumor necrosis factor-α, chemokines, matrix-degrading enzymes, and receptor activator of nuclear factor-κB ligand. These mediators enhance osteoclast differentiation and survival, impair osteoblast function, suppress osteogenic differentiation of bone marrow mesenchymal stromal cells, and propagate paracrine senescence within the bone marrow niche. Senolytics are pharmacological agents that selectively induce apoptosis in senescent cells by targeting senescent-cell anti-apoptotic pathways. Preclinical studies, including those using dasatinib plus quercetin, fisetin, and bone-targeted delivery platforms, have demonstrated the capacity to reduce global senescent-cell burden, attenuate SASP-related inflammation, and improve certain skeletal parameters in models of aging, postmenopausal osteoporosis, and radiotherapy-associated bone loss. However, it remains unclear whether these benefits arise from the elimination of immune cells with senescence-associated features, stromal cells, osteocytes, or a combination thereof. Critically, direct evidence that currently available senolytics selectively eliminate senescence-associated immune-cell subsets within the osteoporotic bone marrow is lacking. Early clinical evidence supports feasibility and hypothesis generation but does not yet establish senolytics as routine osteoporosis therapy. This narrative review synthesizes the mechanistic links among immunosenescence, inflammaging, immune cells with senescence-associated features, and bone remodeling imbalance; critically evaluates current preclinical and clinical evidence for senolytics in osteoporosis; and proposes a structured research roadmap—centered on fate-mapping, lineage-specific depletion, and single-cell multi-omics—to convert the prevailing hypothesis into experimentally testable and clinically actionable evidence, while delineating the translational challenges that must be overcome to achieve precision senolytic therapy.

## 1 Introduction

Osteoporosis is a common age-related skeletal disorder characterized by low bone mass, deterioration of bone microarchitecture, and increased susceptibility to fragility fractures (1, 2). Osteoporotic fractures are associated with pain, disability, loss of independence, increased mortality, and substantial health-care costs. Current pharmacological management includes antiresorptive agents, such as bisphosphonates and denosumab; anabolic agents, such as teriparatide and abaloparatide; and dual-action agents, such as romosozumab (3–6). These treatments have proven clinical benefit, particularly in patients at high or very high fracture risk. Nevertheless, they do not fully reverse age-associated deterioration of the bone marrow microenvironment, the complex cellular and acellular milieu within bone marrow that supports hematopoiesis and skeletal homeostasis, nor do they directly target fundamental mechanisms of skeletal aging. Therefore, strategies that address aging-related drivers of bone remodeling imbalance, i.e., the uncoupling of osteoclast-mediated bone resorption and osteoblast-mediated bone formation that leads to net bone loss, are increasingly being explored.

Bone remodeling is tightly regulated by interactions among osteoclasts, osteoblasts, osteocytes, bone marrow stromal cells, vascular cells, and immune cells. The field of osteoimmunology has demonstrated that the immune and skeletal systems are functionally interconnected, with immune cells and immune-derived mediators directly regulating osteoclastogenesis, osteoblast differentiation, and bone marrow niche homeostasis (7–10). T cells and B cells can modulate bone remodeling through receptor activator of nuclear factor-κB ligand (RANKL), osteoprotegerin (OPG), inflammatory cytokines, and direct cell-cell interactions (10–13). In particular, Th17 cells promote osteoclastogenesis through interleukin-17 (IL-17), RANKL, tumor necrosis factor-α (TNF-α), and related inflammatory pathways, whereas regulatory T cells generally exert anti-osteoclastogenic and immunoregulatory effects (14, 15).

With aging, the immune system undergoes profound remodeling, collectively referred to as immunosenescence. This process includes thymic involution, reduced naïve T-cell output, restricted T-cell receptor diversity, accumulation of late-differentiated memory T cells, impaired B-cell responses, altered innate immune function, and defective immune surveillance. In parallel, aging is accompanied by chronic, sterile, low-grade inflammation, termed inflammaging. Inflammaging is sustained by senescent cells, mitochondrial dysfunction, persistent DNA damage responses, dysregulated innate immune sensing, clonal hematopoiesis, altered gut microbiota, and impaired resolution of inflammation (16–20). These aging-related immune alterations are increasingly recognized as important contributors to osteoporosis.

Cellular senescence is a stress response characterized by stable cell-cycle arrest, resistance to apoptosis, metabolic reprogramming, chromatin remodeling, DNA damage signaling, and acquisition of a senescence-associated secretory phenotype (SASP). The SASP contains cytokines, chemokines, growth factors, proteases, extracellular matrix components, and bioactive lipids that can affect neighboring cells through paracrine mechanisms. In bone, senescent cells accumulate with aging and contribute to increased osteoclast activity, impaired osteoblast function, reduced osteogenic differentiation of bone marrow mesenchymal stromal cells, increased marrow adiposity, and compromised bone strength. Importantly, immune cells are both regulators and targets of senescence. Senescent or senescence-like immune cells may amplify inflammaging and create a bone marrow microenvironment that favors bone resorption over bone formation.

Senolytics are agents designed to selectively eliminate senescent cells by targeting senescent-cell anti-apoptotic pathways. Representative senolytic or senotherapeutic candidates include dasatinib plus quercetin, navitoclax, fisetin, and FOXO4-DRI. In skeletal aging models, senolytic interventions have shown beneficial effects on bone mass, microarchitecture, marrow adiposity, and bone turnover. However, the field remains in an early translational stage. Most studies have examined global senescent-cell burden rather than senescent immune-cell subsets specifically, and clinical data in osteoporosis remain limited.

Rather than providing a systematic review, this article aims to offer a narrative synthesis and conceptual framework for understanding how senescent immune cells may contribute to inflammaging-driven osteoporosis and how senolytic strategies could be developed for skeletal aging. Unlike previous reviews focusing broadly on cellular senescence in bone, this review emphasizes senescent or senescence-like immune cells as a mechanistic bridge between immunosenescence, inflammaging, and osteoporosis, and discusses how this perspective may guide the development of precision senolytic strategies. Concurrently, we critically evaluate the translational challenges, including the lack of cell-type selectivity, the potential for on-target off-tissue toxicity, and the risk of impairing the physiological roles of senescent cells.

## 2 Narrative review approach

This narrative review was developed to summarize current concepts linking immunosenescence, inflammaging, senescent immune cells, and osteoporosis, with a focus on the therapeutic potential and translational challenges of senolytics. Relevant literature was identified through searches of PubMed, Web of Science, and Google Scholar up to May 2026 using combinations of the following terms: “osteoporosis,” “bone remodeling,” “osteoimmunology,” “immunosenescence,” “inflammaging,” “cellular senescence,” “senescent immune cells,” “senescence-associated secretory phenotype,” “T-cell senescence,” “macrophage senescence,” “neutrophil aging,” “B-cell immunosenescence,” “senolytics,” “dasatinib,” “quercetin,” “fisetin,” “navitoclax,” “FOXO4-DRI,” and “bone-targeted senolytics.” Priority was given to PubMed-indexed original studies, clinical trials, mechanistic studies, and high-quality reviews directly relevant to immune aging, skeletal aging, cellular senescence biology, and osteoporosis. Because this article is a narrative review, no formal systematic search protocol, risk-of-bias assessment, or meta-analysis was performed.

## 3 Immunosenescence and inflammaging as emerging mechanisms of osteoporosis

To systematically define these interconnected aging-related processes—immunosenescence, inflammaging, cellular senescence, and the SASP—and to clarify their relevance to skeletal homeostasis, **Table 1** consolidates the core concepts and their characteristics in the context of osteoporosis.

**Table 1:** Core concepts and definitions.

Concept	Definition/Characteristics	Relevance to osteoporosis	References
Osteoporosis	An age-related skeletal disorder characterized by low bone mass, microarchitectural deterioration, and increased fracture risk.	The disease itself, resulting from bone remodeling imbalance.	(1, 2)
Immunosenescence	Age-related remodeling and functional decline of both innate and adaptive immunity, e.g., thymic involution, reduced naïve T-cell output, accumulation of memory T cells.	Creates a chronic inflammatory milieu, predisposing to skeletal aging.	(16–20)
Inflammaging	Chronic, sterile, low-grade inflammation that accompanies aging.	Drives bone resorption and impairs bone formation via pro-inflammatory cytokines; bridges immunosenescence and osteoporosis.	(16–20)
Cellular Senescence	A stress response characterized by stable cell-cycle arrest, apoptosis resistance, metabolic reprogramming, chromatin remodeling, DNA damage signaling, and acquisition of the senescence-associated secretory phenotype (SASP).	Senescent cells accumulate in bone and disrupt homeostasis through SASP or SASP-like factors.	(36–43)
Senescence-Associated Secretory Phenotype (SASP)	The set of pro-inflammatory cytokines, chemokines, growth factors, proteases, and other bioactive molecules secreted by senescent cells. When produced by cells not validated as classically senescent, the terms “SASP-like” or “pro-inflammatory secretome” are more appropriate.	SASP or SASP-like factors (e.g., IL-6, TNF-α) are key mediators that may promote osteoclastogenesis and suppress osteoblast function.	(36–44)
Senolytics	Pharmacological agents that selectively induce apoptosis in senescent cells by targeting senescent-cell anti-apoptotic pathways.	Aim to clear senescent cells from the bone microenvironment, reduce inflammation, and potentially restore bone remodeling balance.	(63–69)

This table consolidates the conceptual framework from the review, defining the core concepts of osteoporosis, immunosenescence, inflammaging, cellular senescence, senescence-associated secretory phenotype, and senolytics, and illustrates how immunosenescence and inflammaging are linked to skeletal aging, and how cellular senescence and the SASP/SASP-like factors may contribute to bone remodeling imbalance, thereby providing the rationale for senolytic strategies in osteoporosis.

SASP, senescence-associated secretory phenotype; RANKL, receptor activator of nuclear factor-κB ligand; OPG, osteoprotegerin; IL-6, interleukin-6; TNF-α, tumor necrosis factor-α.

### 3.1 Definition and characteristics of immunosenescence

Immunosenescence refers to age-related remodeling and functional decline of both innate and adaptive immunity. In the adaptive immune compartment, thymic involution reduces the output of naïve T cells, while chronic antigenic stimulation promotes the accumulation of memory, effector memory, and late-differentiated T-cell subsets (16–20). These changes reduce immune repertoire diversity and impair responses to new antigens. B-cell aging is characterized by reduced generation of naïve B cells, impaired class-switch recombination, decreased somatic hypermutation, and expansion of inflammatory age-associated B-cell phenotypes (21). Innate immune cells also undergo age-related dysfunction, including impaired phagocytosis, altered chemotaxis, defective antigen presentation, dysregulated inflammasome activation, and abnormal cytokine production (21–24).

Not all aged immune cells fulfill the strict definition of classical cellular senescence. Therefore, the term “senescence-like immune cells” may be more appropriate for certain populations, particularly terminally differentiated T cells expressing CD57 or killer cell lectin-like receptor G1 (KLRG1), or lacking the co-stimulatory receptor CD28. Nevertheless, many aged immune cells share features with senescent cells, including reduced proliferative potential, increased DNA damage, telomere attrition, mitochondrial dysfunction, elevated reactive oxygen species, expression of p16^INK4a^ or p21^CIP1^, apoptosis resistance, and secretion of pro-inflammatory mediators (25–28). These changes contribute to immune dysfunction and chronic tissue inflammation. Based on the current literature, this review adopts the following working definition for a ‘senescence-like’ immune cell in the context of the bone marrow: a cell that displays (i) a stable, cell cycle-arrested state (or significantly reduced proliferative capacity); (ii) phenotypic markers associated with aging/dysfunction (e.g., CD28^-^CD57^+^ for T cells); (iii) evidence of DNA damage response or telomere dysfunction; and (iv) a pro-inflammatory secretory phenotype. It is critical to note that no single immune cell subset uniformly displays all these features. This definition serves as a conceptual framework to evaluate the evidence, rather than a diagnostic criterion, and we explicitly acknowledge the ongoing debate regarding whether these cells represent ‘true’ senescence as defined in fibroblasts or epithelial cells.

Given the heterogeneity of immune cell populations and the varying strength of evidence for classical senescence among them, this review adopts a tiered classification framework to more precisely characterize the immune populations discussed. Cells may be categorized as: (i) classically senescent immune cells, which meet all four criteria with multi-marker validation—though such evidence remains limited in osteoporotic bone marrow and none of the populations discussed in this review have been conclusively validated as such; (ii) late-differentiated T cells, defined by CD28^-^CD57^+^/KLRG1^+^ phenotype with proliferative arrest and SASP-like cytokine secretion, but not formally validated as classically senescent; (iii) aged/dysfunctional innate immune cells, such as aged neutrophils, which exhibit functional alterations including impaired phagocytosis, altered chemotaxis, and increased NET formation without meeting classical senescence criteria; and (iv) pro-inflammatory/age-associated immune cells, such as M1-like macrophages and age-associated B cells, which are characterized by inflammatory cytokine production without evidence of cell-cycle arrest. This framework is applied consistently throughout the review when discussing each immune population, and the specific category assignment for each cell type is detailed in Sections 3.3-3.6 and summarized in **Table 2**.

**Table 2:** Senescence-associated features of immune cell subsets and their potential roles in osteoporosis.

Immune cell subset	Senescence-associated features	Potential mechanisms affecting bone remodeling	References
Late-Differentiated T Cells (CD28^-^CD57^+^/KLRG1^+^)	Loss of CD28, expression of CD57 or KLRG1; reduced proliferative capacity, apoptosis resistance. These markers define late-differentiated states that often, but not always, co-occur with features of senescence; they should not be considered synonymous with classical cellular senescence.	May produce RANKL, TNF-α, IL-17, etc., potentially promoting osteoclastogenesis; Th17/Treg imbalance may favor a pro-inflammatory state.	(10–15, 25–28)
Pro-Inflammatory Macrophages (M1-like)	Shift toward an M1-like phenotype; mitochondrial dysfunction, impaired efferocytosis, and phagocytosis. The concept of a “senescent macrophage” is controversial; many of these phenotypes may reflect chronic inflammatory activation rather than classical cellular senescence.	May produce SASP-like inflammatory mediators (TNF-α, IL-1β, IL-6) that could enhance osteoclast differentiation, suppress osteoblasts, and potentially induce stromal cell senescence. Impaired efferocytosis may promote osteoclastogenesis via DAMP signaling.	(29–32)
Aged Neutrophils	Altered chemokine receptor expression, enhanced oxidative burst, increased NET formation. Neutrophil senescence differs fundamentally from classical cellular senescence; NETosis is not a senescence-specific process.	Neutrophil extracellular traps (NETs) may promote osteoclastogenesis and amplify local inflammation and tissue damage.	(21, 23, 24, 33–35)
Age-Associated B Cells (ABCs, CD11b^+^CD21^-^CD23^-^)	Reduced naïve B cells, expansion of inflammatory age-associated B-cell phenotypes. Designated as age-associated B cells to reflect age-related phenotypic changes without implying classical senescence.	May produce RANKL and inflammatory cytokines that could promote osteoclastogenesis; OPG production may be impaired or unbalanced.	(10, 12, 22)

This table consolidates mechanistic evidence from osteoimmunology and immunosenescence research, demonstrating how distinct immune-cell subsets with senescence-associated features—including late-differentiated T cells, pro-inflammatory macrophages, aged neutrophils, and age-associated B cells—may contribute to bone remodeling imbalance in osteoporosis through the production of SASP or SASP-like factors and RANKL, thereby potentially promoting osteoclastogenesis, impairing osteoblast function, and inducing stromal-cell senescence. Bold text indicates critical caveats regarding the distinction between senescence-associated features and classical cellular senescence.

CD28, cluster of differentiation 28; KLRG1, killer cell lectin-like receptor G1; CD57, cluster of differentiation 57; Th17, T helper 17 cells; Treg, regulatory T cells; NETs, neutrophil extracellular traps; M1, M1-like macrophages; DAMP, damage-associated molecular pattern; RANKL, receptor activator of nuclear factor-κB ligand; TNF-α, tumor necrosis factor-α; IL-17, interleukin-17; IL-1β, interleukin-1β; IL-6, interleukin-6; SASP, senescence-associated secretory phenotype; OPG, osteoprotegerin; ABCs, age-associated B cells.

### 3.2 Inflammaging and bone remodeling imbalance

Inflammaging links systemic aging to skeletal deterioration. Canonical SASP or SASP-like factors, including interleukin-1β (IL-1β), IL-6, IL-8, TNF-α, CCL2, CXCL1, and matrix metalloproteinases, can directly or indirectly promote bone loss. Of note, many of these mediators can also be produced by activated or dysfunctional immune cells in the absence of classical cellular senescence. In contrast, systemic inflammatory markers such as C-reactive protein reflect inflammatory status but should not be considered core SASP components produced primarily by senescent cells.

**Figure 1:**
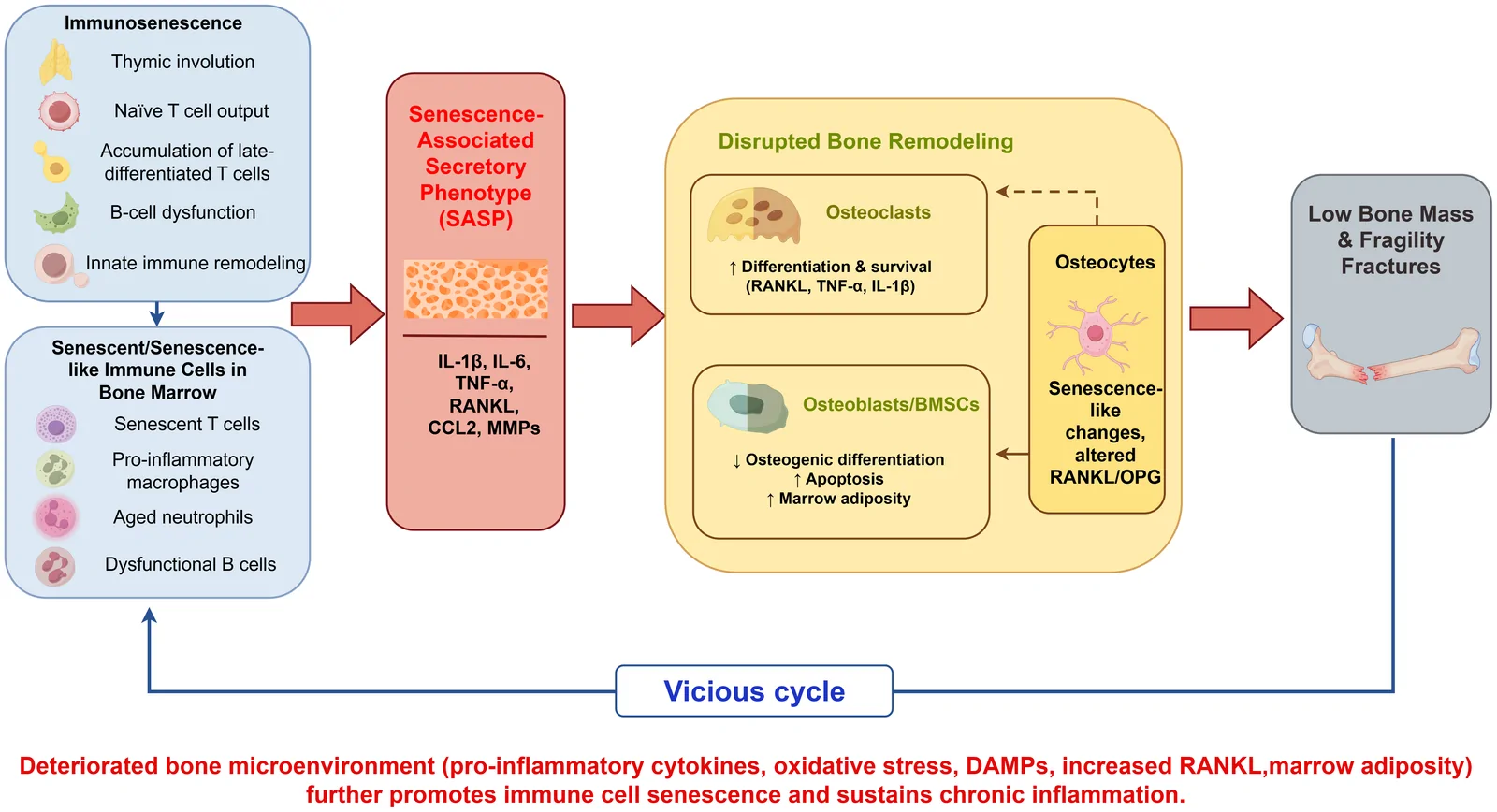
Proposed model of the interplay between immune cells with senescence-associated features, inflammaging, and bone remodeling imbalance in osteoporosis. Aging-driven immune remodeling (immunosenescence) leads to the accumulation of immune cells with senescence-associated features in the bone marrow. These cells produce a pro-inflammatory SASP or SASP-like secretory phenotype enriched in interleukin-1β (IL-1β), IL-6, tumor necrosis factor-α (TNF-α), receptor activator of nuclear factor-κB ligand (RANKL), chemokines, and matrix metalloproteinases. SASP or SASP-like factors may enhance osteoclast differentiation and activity while impairing osteoblast function and the osteogenic differentiation of bone marrow mesenchymal stromal cells (BMSCs). Osteocytes also acquire senescence-like features that disrupt the RANKL/osteoprotegerin (OPG) balance. The resulting uncoupling of bone resorption and formation leads to progressive bone loss and fragility fractures, further reinforcing inflammaging and paracrine senescence within the skeletal microenvironment. The dashed double-headed arrow between “ immune cells with senescence-associated features” and “Secondary senescent stromal cells” indicates a hypothesized, potentially bidirectional, paracrine feedback loop that may amplify inflammaging within the bone marrow niche. Evidence for this loop is currently indirect and requires further validation. SASP, senescence-associated secretory phenotype; IL-1β, interleukin-1β; TNF-α, tumor necrosis factor-α; RANKL, receptor activator of nuclear factor-κB ligand; OPG, osteoprotegerin; BMSCs, bone marrow mesenchymal stromal cells. Figure drawn by www.figdraw.com.

Bone remodeling depends on the balance between osteoclast-mediated bone resorption and osteoblast-mediated bone formation. Inflammatory cytokines promote osteoclast differentiation by increasing expression of RANKL, macrophage colony-stimulating factor (M-CSF), and downstream transcription factors such as nuclear factor of activated T cells cytoplasmic 1 (NFATc1). RANKL binds to RANK on osteoclast precursors, whereas OPG acts as a decoy receptor that inhibits RANKL-RANK interaction. Therefore, the RANKL/OPG ratio is a major determinant of osteoclastogenesis and bone resorption. Inflammatory mediators also suppress osteoblast differentiation by inhibiting Runx2 and Osterix, inducing oxidative stress, impairing Wnt signaling, and reducing the osteogenic capacity of bone marrow mesenchymal stromal cells. The overall mechanistic link from immunosenescence and immune cells with senescence-associated features, through SASP or SASP-like-driven inflammaging, to uncoupled bone remodeling in osteoporosis is summarized in **Figure 1**.

### 3.3 Late-differentiated T cells and their role in osteoporosis

T cells are important regulators of bone metabolism. However, identifying which T-cell subsets in the aging bone marrow are truly ‘senescent’ versus simply ‘activated’ or ‘terminally differentiated’ remains a major challenge (25, 26). The widely used markers CD28 loss and CD57 or KLRG1 expression (25–28) should not be considered synonymous with cellular senescence. Instead, they define late-differentiated states that often, but not always, co-occur with features of senescence such as proliferative arrest and DNA damage. Consistent with the classification framework introduced in Section 3.1, these cells are therefore designated late-differentiated T cells in this review. Evidence derived from studies on CD28^-^CD57^+^ T cells is presented as supportive of, but not definitive proof for, a role of senescence-associated T-cell states in osteoporosis. Activated and late-differentiated T cells can produce RANKL, TNF-α, interferon-γ, IL-17, and other inflammatory mediators, thereby influencing osteoclast formation and activity (10–15). In postmenopausal and age-related osteoporosis, estrogen deficiency and aging-associated immune remodeling can enhance T-cell activation and inflammatory cytokine production. Late-differentiated CD4+ and CD8+ T cells, often marked by CD28 loss and expression of CD57 or KLRG1, may contribute to chronic inflammatory signaling and are thus considered senescence-associated T-cell populations in this review (25–28). However, these markers are not fully specific for cellular senescence and should be interpreted together with functional features such as proliferative arrest, DNA damage, p16^INK4a^ expression, SASP-like cytokine secretion, and apoptosis resistance.

The Th17/Treg balance is particularly relevant to skeletal aging. Th17 cells promote osteoclastogenesis mainly through IL-17 and RANKL-related pathways, whereas regulatory T cells can suppress osteoclast differentiation and dampen inflammatory bone loss (14, 15). Aging-related shifts toward a pro-inflammatory T-cell phenotype may therefore increase osteoclast activity and contribute to impaired bone homeostasis.

### 3.4 Pro-inflammatory macrophages in the bone marrow niche

The concept of a ‘senescent macrophage’ is particularly controversial. Many of the phenotypes attributed to senescent macrophages, such as increased TNF-α, IL-1β, and IL-6, may simply reflect a shift towards a chronic inflammatory M1-like polarization state (29–31), rather than the multi-faceted program of cell-cycle arrest and SASP that defines classical senescence. Consistent with the classification framework in Section 3.1, macrophages discussed in this review are therefore designated as pro-inflammatory macrophages (or M1-like macrophages) rather than senescent macrophages, as there is currently insufficient evidence that these cells undergo classical cellular senescence in the osteoporotic bone marrow. Macrophages participate in both immune defense and bone remodeling. In bone, macrophage-lineage cells contribute to osteoclast formation, regulate osteoblast function, clear apoptotic cells, and support tissue repair. With aging, macrophages may exhibit mitochondrial dysfunction, impaired efferocytosis, the process of clearing apoptotic cells. In the bone marrow niche, efficient efferocytosis by macrophages is crucial for maintaining tissue homeostasis and resolving inflammation. Impaired efferocytosis in aged macrophages could lead to the accumulation of apoptotic cellular debris, which may act as a damage-associated molecular pattern (DAMP) to sustain inflammatory signaling and potentially promote osteoclastogenesis. In addition, aged macrophages often show altered autophagy, reduced phagocytic capacity, and increased production of pro-inflammatory cytokines. Macrophages with senescence-associated features, or those that are dysfunctional, often show a shift toward an M1-like inflammatory phenotype, characterized by increased TNF-α, IL-1β, IL-6, inducible nitric oxide synthase, and NF-κB activation (29–31).

These macrophage-derived mediators can promote osteoclastogenesis and suppress osteoblast differentiation. In addition, macrophage-derived inflammatory mediators may induce senescence-like changes in bone marrow mesenchymal stromal cells and impair their osteogenic differentiation. A recent study showed that Astragaloside IV attenuated macrophage senescence and d-galactose-induced bone loss through the STING/NF-κB pathway, supporting the concept that macrophage senescence-associated phenotypes can influence bone loss through immune-inflammatory mechanisms (32). However, Astragaloside IV should be considered a senescence-modulating compound rather than a classical senolytic unless selective elimination of senescent cells is demonstrated.

### 3.5 Aged neutrophils, NETs, and bone inflammation

Neutrophils are short-lived innate immune cells, and the concept of “neutrophil senescence” differs from classical cellular senescence. Consistent with the classification framework in Section 3.1, neutrophils are designated as aged neutrophils in this review to reflect their functional alterations without implying classical senescence. Aged neutrophils display altered chemokine receptor expression, changes in CXCR2/CXCR4 signaling, delayed apoptosis, enhanced oxidative burst, and increased formation of neutrophil extracellular traps (NETs) (21, 23, 24). NETs contain DNA, histones, and proteases that can amplify tissue damage. It is important to note that while aged neutrophils may have an increased propensity to form NETs, NETosis itself is not a senescence-specific process and can be triggered by various inflammatory stimuli. Therefore, the presence of NETs in the osteoporotic bone marrow should be interpreted as a marker of neutrophil activation and inflammation, which may be exacerbated by the aging process, rather than a direct readout of neutrophil senescence. Emerging studies suggest that NETs may contribute to osteoporosis and inflammatory bone erosion by promoting osteoclastogenesis, impairing osteoblast function, and amplifying local inflammatory signaling (33–35). Nevertheless, direct evidence linking aged neutrophils specifically to senile osteoporosis remains less mature than evidence for T cells and macrophages.

### 3.6 Age-associated B cells and bone remodeling

**Figure 2:**
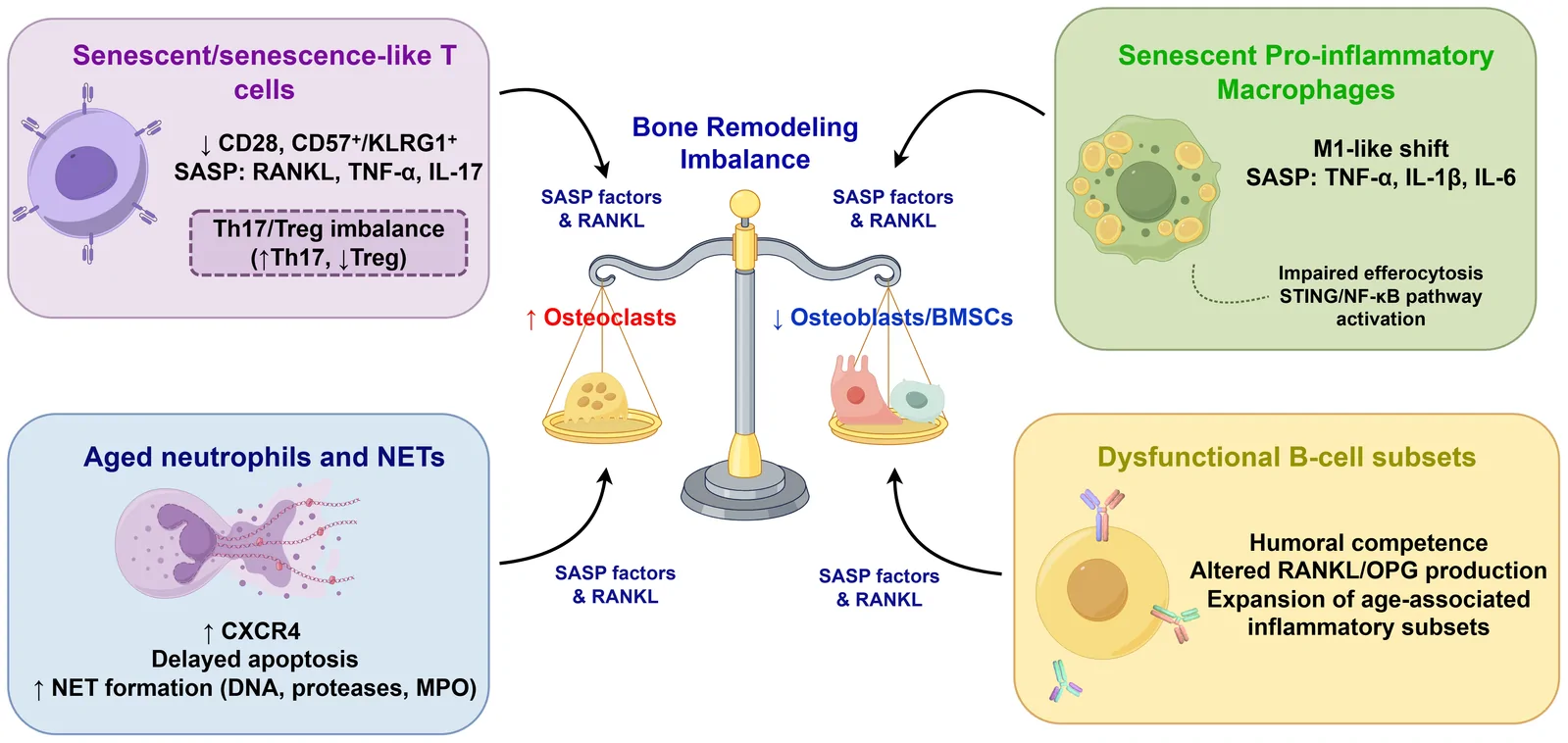
Contribution of late-differentiated T cells, pro-inflammatory macrophages, aged neutrophils, and age-associated B cells to osteoclastogenesis and impaired bone formation. Late-differentiated CD4^+^ and CD8^+^ T cells, often characterized by CD28 loss and expression of CD57 or killer cell lectin-like receptor G1 (KLRG1), may promote osteoclastogenesis through RANKL, TNF-α, and interleukin-17 (IL-17), partly via an imbalance between T helper 17 (Th17) and regulatory T cells. Pro-inflammatory macrophages (M1-like) exhibit increased secretion of TNF-α, IL-1β, and IL-6, and show impaired efferocytosis and activation of the stimulator of interferon genes (STING)/nuclear factor-κB (NF-κB) pathway. Aged neutrophils display delayed apoptosis and enhanced release of neutrophil extracellular traps (NETs), which may amplify osteoclastogenesis and local inflammation. Age-associated B-cell subsets (ABCs) exhibit altered RANKL/OPG production, potentially contributing to a pro-resorptive milieu. Collectively, these immune cell populations may contribute to the disruption of the balance between bone resorption and formation. KLRG1, killer cell lectin-like receptor G1; IL-17, interleukin-17; Th17, T helper 17; STING, stimulator of interferon genes; NF-κB, nuclear factor-κB; NETs, neutrophil extracellular traps. Figure drawn by www.figdraw.com.

B cells influence bone remodeling by producing cytokines, antigen-presenting signals, RANKL, and OPG. Aging is associated with reduced humoral immune competence, impaired vaccine responses, and expansion of inflammatory B-cell subsets. Consistent with the classification framework in Section 3.1, these are designated as age-associated B cells (ABCs, typically CD11b^+^CD21^-^CD23^-^) and plasma cells in this review to reflect their age-related phenotypic changes without implying classical senescence. These populations are known to produce pro-inflammatory cytokines (21, 22). In skeletal disease, B-cell-derived RANKL and cytokines may promote osteoclastogenesis, whereas OPG production can counteract RANKL-mediated resorption (10, 12, 22). Therefore, the net effect of B cells on bone depends on B-cell subset composition, activation state, and local inflammatory context. In osteoporosis research, further work is needed to define whether specific age-associated B-cell subsets function as senescent or senescence-associated immune-cell populations in the bone marrow. Given the diversity of immune populations affected by aging and the complexity of their interactions within the bone marrow niche, **Table 2** summarizes the senescence-associated features of major immune-cell subsets—including late-differentiated T cells, pro-inflammatory macrophages, aged neutrophils, and age-associated B cells—and their respective potential mechanisms contributing to bone remodeling imbalance. The distinct contributions of senescent-like T cells, pro-inflammatory macrophages, aged neutrophils with enhanced NET formation, and dysfunctional B-cell subsets to osteoclastogenesis and impaired bone formation are illustrated in **Figure 2**.

## 4 SASP factor network and its effects on bone cells

### 4.1 Composition of SASP factors relevant to bone

Cellular senescence is characterized by stable cell-cycle arrest, resistance to apoptosis, DNA damage signaling, chromatin remodeling, metabolic changes, and acquisition of the SASP (36–43). The SASP is heterogeneous and varies according to cell type, senescence inducer, tissue context, and disease stage. In the skeletal microenvironment, SASP-related mediators with potential relevance include IL-1β, IL-6, IL-8, TNF-α, CCL2, CXCL1, CXCL8, MMP-1, MMP-3, MMP-9, vascular endothelial growth factor, transforming growth factor-β, and prostaglandins (36–47). While the mediators listed above are classically associated with the SASP of senescent cells, it is important to recognize that many of these factors can also be produced by activated, late-differentiated, or dysfunctional immune cells in the absence of classical cellular senescence. In such contexts, the terms “SASP-like” or “pro-inflammatory secretome” are more appropriate. These factors can recruit immune cells, propagate paracrine senescence, remodel extracellular matrix, and alter osteoblast-osteoclast coupling. To provide a comprehensive overview of how senescence-associated secreted mediators translate into skeletal dysfunction, **Table 3** outlines the major SASP factors relevant to the bone microenvironment and their differential effects on osteoclastogenesis versus osteoblast differentiation and stromal cell function.

**Table 3:** Key SASP/SASP-like factors and their direct effects on bone cells.

SASP/SASP-like factor	Effects on osteoclasts	Effects on osteoblasts/bone marrow mesenchymal stromal cells	References
IL-1β, TNF-α	May directly promote osteoclast differentiation and survival; may enhance RANKL expression, potentially synergistically driving bone resorption. TNF-α can exert concentration-dependent effects on osteoblasts, promoting apoptosis at high doses while potentially activating pro-survival pathways at lower doses.	May suppress osteoblast differentiation (inhibit RUNX2 and other transcription factors), induce oxidative stress and apoptosis.	(44–47)
IL-6	May expand osteoclast precursors via JAK/STAT3 signaling. The role of IL-6 in bone is complex; it can also promote osteoblast differentiation and matrix mineralization under certain conditions.	May inhibit osteoblast differentiation and bone formation in most contexts, though effects may be context-dependent.	(48, 49)
Chemokines (CCL2, CXCL1)	May recruit monocytes/macrophages, potentially increasing the pool of osteoclast precursors.	May recruit inflammatory cells, indirectly aggravating inflammatory bone loss; may also influence osteoblast migration and differentiation in a concentration-dependent manner.	(44)
Matrix Metalloproteinases (MMPs)	May degrade bone matrix, release matrix-sequestered growth factors, perturb remodeling.	May disrupt the extracellular matrix required for osteoblast attachment and function.	(44)

This table consolidates mechanistic evidence from cellular senescence and skeletal biology studies, demonstrating the key SASP factors—including IL-1β, TNF-α, IL-6, chemokines (CCL2, CXCL1), and matrix metalloproteinases—and their dual effects on osteoclasts and osteoblasts/bone marrow mesenchymal stromal cells, thereby elucidating how these mediators promote enhanced bone resorption and impaired bone formation in osteoporosis through enhanced osteoclastogenesis and suppressed osteogenic differentiation. Bold text indicates critical contextual caveats regarding concentration- and cell-type-dependent effects.

SASP, senescence-associated secretory phenotype; IL-1β, interleukin-1β; TNF-α, tumor necrosis factor-α; IL-6, interleukin-6; CCL2, C-C motif chemokine ligand 2; CXCL1, C-X-C motif chemokine ligand 1; MMPs, matrix metalloproteinases; RUNX2, Runt-related transcription factor 2; JAK/STAT3, Janus kinase/signal transducer and activator of transcription 3; NF-κB, nuclear factor-κB.

NF-κB is a central regulator of SASP-related inflammatory signaling and can sustain cytokine and chemokine production in senescent cells (44). IL-6 can activate JAK/STAT3 signaling and contribute to osteoclast precursor expansion and inflammatory bone resorption (48, 49). TNF-α promotes osteoclast differentiation directly and indirectly by increasing RANKL expression in stromal and osteoblastic cells. IL-1β enhances osteoclast survival and activity and can synergize with TNF-α and RANKL (45–47). Chemokines such as CCL2 recruit monocytes and macrophages, increasing the pool of osteoclast precursors and inflammatory effector cells. MMPs degrade extracellular matrix and may release matrix-sequestered growth factors, further disturbing bone remodeling (44).

### 4.2 From SASP to skeletal dysfunction: a question of concentration, context, and cell type

While the list of SASP factors in **Table 3** is extensive, their individual and combined effects on bone cells are highly context-dependent. For instance, TNF-α is a potent activator of the NF-κB pathway in osteoclast precursors and synergizes with RANKL to promote osteoclast differentiation (50–52). However, in osteoblasts, TNF-α can exert dual effects, promoting apoptosis at high concentrations while potentially activating pro-survival pathways such as NF-κB and MAPK at lower doses, thereby exerting context-dependent effects on bone formation. Similarly, the role of IL-6 in bone remodeling is complex; it can stimulate osteoclastogenesis through JAK/STAT3 signaling, but also promotes osteoblast differentiation and matrix mineralization under certain conditions. The chemokine CCL2, while recruiting monocyte/macrophage precursors to sites of bone resorption, can also influence osteoblast migration and differentiation in a concentration-dependent manner. Such nuances are critical; simply stating that “SASP factors promote bone loss” is an oversimplification that obscures the complex and sometimes opposing effects of these mediators. A more refined understanding requires consideration of the specific SASP or SASP-like composition of a given immune subset with senescence-associated features, the local concentration gradient within the bone marrow niche, and the differentiation stage and lineage commitment of the target bone cell.

### 4.3 Effects on osteoclasts

Osteoclasts arise from monocyte/macrophage lineage precursors. Their differentiation requires M-CSF and RANKL, followed by activation of downstream signaling pathways, including NF-κB, MAPK, AP-1, and NFATc1 (50, 51). RANKL-RANK-OPG signaling, NFATc1 activation, and inflammatory mediators such as TNF-α are central mechanisms driving osteoclast differentiation and inflammatory bone resorption (50–52). SASP factors can enhance osteoclastogenesis by increasing RANKL production, sensitizing precursors to RANKL, prolonging osteoclast survival, and sustaining inflammatory signaling. TNF-α and IL-1β are particularly important in inflammatory bone loss, as they enhance osteoclast differentiation and activity. Therefore, immune cells with senescence-associated features may accelerate bone resorption by providing both osteoclastogenic cytokines and precursor-recruiting chemokines.

### 4.4 Effects on osteoblasts and bone marrow mesenchymal stromal cells

Osteoblasts and bone marrow mesenchymal stromal cells are highly sensitive to inflammatory and senescence-associated signals. SASP or SASP-like factors can suppress osteogenic differentiation, impair mineralization, induce oxidative stress, and promote apoptosis or senescence in osteoblast-lineage cells. Senescent-cell conditioned media have been shown to inhibit osteoblast mineralization and alter osteoclast precursor survival, suggesting that senescent cells can disrupt bone remodeling through paracrine mechanisms (53–55). Moreover, aging and inflammatory signaling favor adipogenic differentiation of bone marrow stromal cells, contributing to marrow adiposity and reduced osteogenic potential (56–61).

### 4.5 Paracrine propagation of senescence in the bone marrow microenvironment

**Figure 3:**
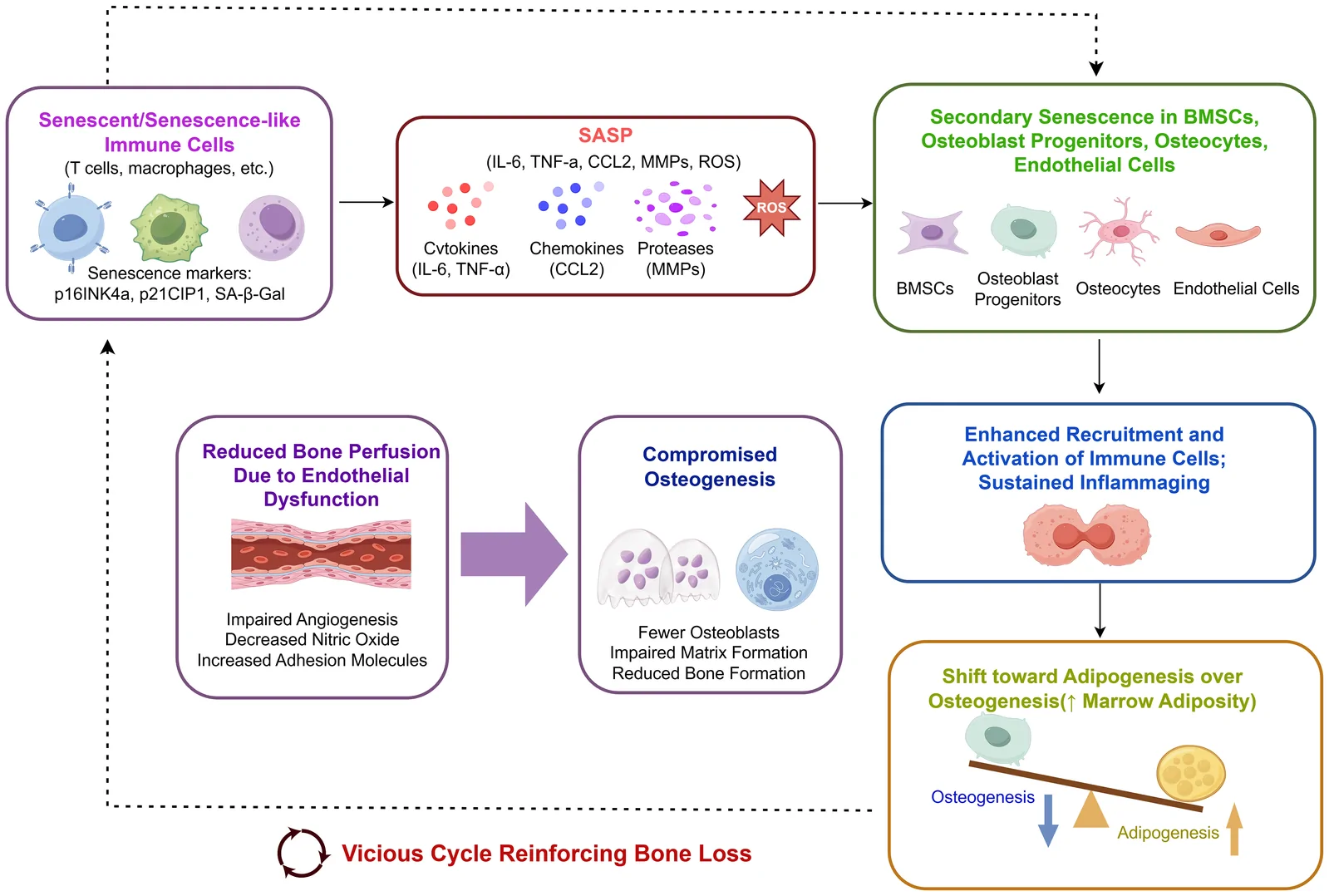
Conceptual model of the self-amplifying senescent-inflammatory niche in the bone marrow. Immune cells with senescence-associated features produce a pro-inflammatory SASP or SASP-like secretory phenotype enriched in IL-1β, IL-6, TNF-α, chemokines, and matrix metalloproteinases. The SASP or SASP-like factors can induce secondary senescence in neighboring cells, including bone marrow mesenchymal stromal cells (BMSCs), osteoblast progenitors, osteocytes, and endothelial cells (these are proposed candidate cell types for secondary senescence; however, direct *in vivo* evidence for this specific sequence remains preliminary and requires further validation). These newly senescent cells secrete additional SASP or SASP-like factors, recruit further immune cells, and promote adipogenic differentiation at the expense of osteogenesis, leading to increased marrow adiposity. Endothelial dysfunction further impairs bone perfusion, compromising nutrient supply and osteogenesis. The dashed arrows represent hypothesized paracrine effects that are supported primarily by *in vitro* or indirect evidence, and the entire loop should be viewed as a conceptual framework rather than a fully established pathway. Importantly, BMSCs, osteoblast progenitors, osteocytes, and endothelial cells may also represent primary sources of senescence that arise independently of immune-cell-derived signals and secondarily recruit or activate immune cells. The relative contributions of primary versus secondary senescence to the establishment and maintenance of this niche remain to be determined. This self-amplifying inflammatory niche sustains and accelerates age-related bone loss. SASP, senescence-associated secretory phenotype; BMSCs, bone marrow mesenchymal stromal cells; IL-1β, interleukin-1β; IL-6, interleukin-6; TNF-α, tumor necrosis factor-α. Figure drawn by www.figdraw.com.

Senescent or senescence-like immune cells do not act in isolation. Their SASP or SASP-like inflammatory mediators can induce senescence-like changes in neighboring stromal cells, endothelial cells, osteoblast progenitors, osteocytes, and hematopoietic cells. This suggests a potential self-amplifying inflammatory niche in the bone marrow. Endothelial dysfunction and impaired angiogenesis may further reduce bone perfusion and compromise osteogenesis. Senescent stromal cells, in turn, produce additional SASP factors that recruit immune cells and sustain inflammation. In addition to these paracrine effects, autocrine SASP signaling may further reinforce the senescent phenotype within individual cells, creating a self-sustaining cycle of inflammation and dysfunction. Thus, senescence-associated changes in immune cells and stromal cells may reinforce each other during skeletal aging. However, it is currently unclear whether this represents a unidirectional process initiated by immune cells, or a bidirectional vicious cycle in which stromal and immune cell senescence are mutually reinforcing. The evidence for secondary senescence of bone marrow mesenchymal stromal cells, osteoblast progenitors, and osteocytes is derived primarily from *in vitro* experiments or indirect *in vivo* observations, and definitive *in vivo* proof that senescent or senescence-like immune cells are the primary inducers of secondary senescence in the bone marrow niche is lacking. This remains a central, testable hypothesis for future research. The possibility that bone marrow mesenchymal stromal cells, osteocytes, and endothelial cells may themselves represent primary sources of senescence that secondarily recruit and activate immune cells should also be considered. This self-amplifying inflammatory loop, in which senescent or senescence-like immune cells and secondary senescent stromal/osteoblast-lineage cells may reinforce each other, is depicted in **Figure 3**. Of note, the candidate secondary senescent cell types depicted in **Figure 3**—BMSCs, osteoblast progenitors, osteocytes, and endothelial cells—have been identified primarily through *in vitro* studies or indirect *in vivo* observations (53–57, 62). These cell populations may also undergo primary senescence independently of immune-cell-derived signals, and their relative contributions to the establishment and maintenance of the senescent-inflammatory niche remain an important area for future investigation.

## 5 Cellular senescence in the bone microenvironment

### 5.1 Identification of senescent cells in bone

Senescent cells accumulate in the bone microenvironment with aging, including osteocytes, osteoblast-lineage cells, myeloid-lineage cells, stromal cells, and marrow adipocyte-related populations. These cells are commonly identified by a combination of markers, including p16^INK4a^, p21^CIP1^, senescence-associated β-galactosidase activity, DNA damage markers, telomere-associated foci, SASP factor expression, and resistance to apoptosis (53–57). No single marker is sufficient to define senescence *in vivo*, particularly in complex tissues such as bone marrow. Therefore, studies of skeletal senescence increasingly rely on multi-marker strategies and cell-type-specific approaches.

### 5.2 Senescent osteocytes and osteoblast-lineage cells

Osteocytes are long-lived cells embedded in the mineralized bone matrix and act as major regulators of bone remodeling through sclerostin, RANKL, OPG, and mechanosensory signaling. Osteocytes and osteoblast-lineage cells have been suggested as candidate cell types for secondary senescence, although direct *in vivo* evidence for this is still emerging. With aging, osteocytes may acquire senescence-like features and contribute to altered bone remodeling. Senescent osteocytes and osteoblast-lineage cells can secrete inflammatory cytokines and matrix-modifying enzymes, thereby influencing both osteoclastogenesis and osteoblast function. These cells may also communicate with bone marrow immune cells and stromal cells, further integrating skeletal senescence with inflammaging. Whether osteocyte senescence *in vivo* is primarily driven by paracrine SASP from neighboring immune cells, or by cell-intrinsic aging mechanisms such as telomere attrition and accumulated DNA damage, remains an open question (56, 62).

### 5.3 Senescence, marrow adiposity, and skeletal aging

Aging bone marrow is characterized by increased adiposity and reduced osteogenic potential. Senescence-related mechanisms contribute to this shift by impairing osteogenic differentiation of stromal cells and favoring adipogenic lineage commitment. Bone marrow mesenchymal stromal cells have been proposed as candidate targets of secondary senescence, although definitive *in vivo* evidence for their role as secondary responders to senescent immune cells remains limited. Studies of radiation- and aging-related bone loss indicate that marrow adiposity is linked to cellular senescence (60, 61). Increased marrow adiposity may further impair bone formation through local lipotoxicity, adipokine production, oxidative stress, and inflammatory signaling. Therefore, senescent cells may contribute to osteoporosis not only by increasing bone resorption but also by remodeling the marrow niche in a manner unfavorable to osteogenesis. The extent to which marrow adiposity is a consequence of secondary senescence induced by immune-cell SASP or SASP-like factors, versus a primary senescent phenotype of mesenchymal stromal cells themselves, warrants further investigation using lineage-tracing and cell-type-specific senescence models (59–61).

### 5.4 Cellular senescence as a therapeutic target in osteoporosis

Cellular senescence has been increasingly recognized as a causal contributor and therapeutic target in age-related osteoporosis (54, 55, 57–59). Genetic and pharmacological studies indicate that reducing senescent-cell burden can prevent or attenuate age-related bone loss. However, skeletal senescence is cell-type heterogeneous, and the relative contributions of senescent or senescence-like immune cells, osteocytes, osteoblast-lineage cells, stromal cells, endothelial cells, and adipocyte progenitors remain incompletely understood. This heterogeneity has important therapeutic implications because different senescent-cell populations may depend on distinct survival pathways and may respond differently to senolytic interventions.

## 6 Pharmacological mechanisms of senolytics and senomorphic strategies

### 6.1 Definition of senolytics

Senolytics are agents that selectively induce apoptosis in senescent cells while sparing most non-senescent cells. Senescent cells survive despite cellular damage by upregulating senescent-cell anti-apoptotic pathways, including BCL-2 family proteins, PI3K/AKT signaling, p53/p21-related survival mechanisms, ephrin receptor signaling, hypoxia-inducible factor pathways, and pro-survival tyrosine kinase signaling (63–69). Senolytics exploit these dependencies to lower senescent-cell burden. In contrast, senomorphics (or senostatics) are compounds that suppress the harmful SASP without necessarily eliminating senescent cells.

### 6.2 Representative senolytic agents

Dasatinib plus quercetin is one of the earliest and most widely studied senolytic combinations. Dasatinib is a tyrosine kinase inhibitor, whereas quercetin is a flavonoid that affects PI3K, serpines, and other survival pathways. Their combination was identified as having complementary activity across different senescent-cell types (63). Navitoclax is a BCL-2/BCL-xL inhibitor that can eliminate certain senescent-cell populations but is limited by toxicity, especially thrombocytopenia, and by context-dependent skeletal effects (64). Fisetin, another natural flavonoid, has senotherapeutic activity in multiple preclinical models and can reduce senescence markers *in vivo* (65). FOXO4-DRI is a peptide designed to disrupt FOXO4-p53 interaction, thereby promoting apoptosis of senescent cells in experimental models (66). Collectively, these agents illustrate the diversity of senolytic mechanisms and the need for cell-type-specific evaluation.

In osteoporosis, the most relevant senolytic evidence currently comes from D+Q, fisetin, and bone-targeted delivery platforms. However, not all compounds labeled as senolytics have equivalent evidence for skeletal efficacy, immune-cell specificity, or clinical safety. Therefore, senolytic candidates should be evaluated according to their mechanism of action, senescent-cell selectivity, target tissue distribution, pharmacokinetics, toxicity profile, and disease-specific efficacy.

### 6.3 The hypothesis and challenge of selectively targeting senescence-associated immune cells

Senescent or senescence-like immune cells may be vulnerable to senolytic strategies because they often show apoptosis resistance and increased expression of pro-survival pathways. Nevertheless, direct evidence that senolytics selectively eliminate senescent T cells, macrophages, neutrophils, or B cells in osteoporotic bone marrow remains limited. Most available skeletal studies report reductions in global senescent-cell markers, such as p16^INK4a^, p21^CIP1^, senescence-associated β-galactosidase activity, and SASP factors, rather than immune-subset-specific senolysis (54, 55, 60, 61, 70). Importantly, these global marker reductions could result from the elimination of senescent osteocytes, bone marrow mesenchymal stromal cells, endothelial cells, or adipocyte progenitors, rather than from the selective depletion of immune-cell subsets (56, 62). Furthermore, as discussed in Sections 3.3-3.6, the senescence-like states of T cells, macrophages, neutrophils, and B cells are defined primarily by phenotypic markers and functional characteristics, many of which overlap with chronic activation or polarization states. It remains unclear whether these cells depend on the canonical senescent-cell anti-apoptotic pathways—such as BCL-2 family proteins, PI3K/AKT signaling, or ephrin receptor signaling—that are targeted by current senolytics (63–66). Therefore, claims about senolytics targeting senescent or senescence-like immune cells should be presented as a plausible mechanism that requires further validation, rather than as an established therapeutic principle. Future studies employing lineage-specific senescence models, single-cell transcriptomics, and *in vivo* fate-mapping approaches will be essential to determine whether senolytic agents can indeed eliminate specific senescence-associated immune populations and whether such elimination contributes meaningfully to the preservation of skeletal health.

### 6.4 Senolytic versus senomorphic effects

Many compounds used in osteoporosis models have antioxidant, anti-inflammatory, osteogenic, or antiresorptive effects in addition to possible senolytic activity. For example, natural flavonoids may suppress oxidative stress and inflammatory pathways independently of senescent-cell clearance. Therefore, it is essential to distinguish true senolysis from SASP suppression, immune polarization, osteoblast stimulation, or osteoclast inhibition. Future studies should include direct assays of senescent-cell apoptosis, lineage-specific senescent-cell depletion, and rescue experiments to confirm mechanism.

### 6.5 Safety considerations and the double-edged sword of senolysis

A distinctive feature of many senolytic regimens is intermittent dosing. Because senescent cells do not need continuous drug exposure to undergo apoptosis, intermittent administration may reduce toxicity compared with chronic treatment (67–69). However, safety remains a major translational challenge. Navitoclax can cause thrombocytopenia due to BCL-xL inhibition in platelets (64). Dasatinib may cause myelosuppression, fluid retention, pleural effusion, and drug interactions, although short intermittent dosing may have a different risk profile from chronic oncology dosing. Natural flavonoids such as quercetin and fisetin are generally perceived as safer but still require rigorous pharmacokinetic, dosing, and interaction studies.

In addition to drug-specific toxicities like thrombocytopenia with navitoclax (64), there are fundamental biological risks that are intrinsic to the senolytic approach itself. Senescent cells play beneficial roles in wound healing, tissue repair, embryonic development, and as a potent anti-tumor mechanism (71–73). Non-selective or chronic clearance could therefore impair these essential physiological processes. This is particularly concerning in the frail, elderly osteoporotic population, where comorbidities, polypharmacy, immune vulnerability, impaired healing capacity, and reduced physiological reserve are common. The navitoclax example (Section 7.4) perfectly illustrates this point, showing that in the bone microenvironment itself, senescent-cell clearance can be detrimental under certain conditions. This finding challenges the assumption that senescent-cell removal is universally protective and underscores the need for a more nuanced understanding of the context-dependent effects of senolysis. Therefore, any clinical development program must incorporate rigorous monitoring for delayed wound healing, increased infection rates, and long-term cancer risk, in addition to standard hematologic and metabolic safety assessments.

## 7 Preclinical evidence of senolytics in osteoporosis and skeletal aging

### 7.1 Targeting cellular senescence in age-related bone loss

A landmark study demonstrated that genetic and pharmacological targeting of senescent cells prevented age-related bone loss in mice. In that study, senescent-cell clearance reduced bone resorption while maintaining or improving bone formation, leading to better bone mass, microarchitecture, and strength. Mechanistically, senescent-cell conditioned media impaired osteoblast mineralization and supported osteoclast precursor survival, suggesting that senescent cells contribute to bone loss through secreted factors (54). This work established cellular senescence as a therapeutic target in skeletal aging.

### 7.2 D+Q in postmenopausal and aging-related models

The combination of dasatinib and quercetin has been evaluated in several preclinical skeletal models. In postmenopausal osteoporosis-related models, D+Q reduced senescence markers, improved bone regeneration, and attenuated bone loss (70). In radiotherapy-associated bone loss, targeted reduction of senescent-cell burden alleviated skeletal deterioration and improved bone parameters (60). D+Q has also been linked to reduced marrow adiposity in radiation- and aging-related bone loss models, suggesting that senescent cells may influence the balance between osteogenesis and adipogenesis within the bone marrow niche (61).

However, interpretation should remain cautious. These studies support senescent-cell targeting in bone but do not always distinguish whether the primary targets are senescent or senescence-like immune cells, osteocytes, osteoblast-lineage cells, stromal cells, endothelial cells, or adipocyte progenitors. Future studies should use single-cell transcriptomics, lineage tracing, and immune-cell-specific senescence models to clarify cell-type-specific effects.

### 7.3 Fisetin and other natural senotherapeutics

Fisetin has demonstrated broad senotherapeutic activity in aging models (65). In osteoporosis-related experimental models, fisetin has been reported to reduce osteoclast activity, improve bone microarchitecture, and modulate oxidative stress and RANKL/OPG signaling (74). These findings are promising, but more rigorous comparative studies are needed to determine whether fisetin acts primarily as a senolytic, senomorphic, antioxidant, anti-inflammatory agent, or a combination of these mechanisms in bone.

Astragaloside IV has shown effects on macrophage senescence and d-galactose-induced bone loss through STING/NF-κB signaling (32). This finding is particularly relevant to the theme of senescent or senescence-like immune cells. However, because Astragaloside IV appears to modulate macrophage senescence and polarization rather than selectively eliminate senescent cells, it should be discussed as a senescence-modulating or senomorphic candidate rather than a classical senolytic.

### 7.4 Navitoclax: potential and caution

Navitoclax (ABT-263) is a potent inhibitor of BCL-2, BCL-xL, and BCL-w that can eliminate certain senescent-cell populations by disrupting the anti-apoptotic survival pathways upon which these cells depend (64). Preclinically, navitoclax has demonstrated senolytic activity in multiple tissues, including hematopoietic system, lung, and adipose tissue, and has been shown to reduce senescence markers and improve age-related phenotypes in various mouse models (64, 67). However, its effects in bone appear to be highly context-dependent and serve as a critical cautionary tale for the field of skeletal senolytics. In aged mice, short-term navitoclax treatment unexpectedly caused trabecular bone loss and impaired osteoprogenitor function, suggesting that not all senolytic agents are beneficial for bone under all conditions (75). This finding is of paramount importance because it demonstrates that senescent-cell clearance is not uniformly anabolic in the skeleton. Rather, the net effect of senolysis depends on the specific cell types eliminated, the timing of administration relative to the skeletal and inflammatory state, and the baseline bone turnover status. The adverse skeletal effects of navitoclax are thought to be related to its inhibition of BCL-xL, which may play a role in osteoprogenitor survival and function, although direct mechanistic evidence for this specific effect in bone requires further investigation. This mechanistic insight underscores the dangers of a non-selective approach to senescent-cell clearance, particularly when the target cell populations—whether immune cells, stromal cells, or osteoblast-lineage cells—share survival pathways with cells that are critical for maintaining skeletal homeostasis. Moreover, navitoclax is associated with significant on-target hematologic toxicity, most notably dose-limiting thrombocytopenia due to BCL-xL inhibition in platelets, which further complicates its translational potential in the frail, elderly osteoporotic population. Therefore, navitoclax should be discussed cautiously in the context of osteoporosis. Its bone phenotype serves as a powerful reminder that the hypothesis that “senolytics protect bone by clearing senescent immune cells” requires rigorous cell-type-specific testing and that senolytic efficacy cannot be assumed based solely on the ability to reduce global senescence markers. Any clinical development of navitoclax or related BCL-2 family inhibitors for skeletal indications would require strategies to mitigate osteoprogenitor toxicity and thrombocytopenia, such as bone-targeted delivery, intermittent dosing schedules, or the development of more selective inhibitors that spare BCL-xL in critical cell types.

### 7.5 Bone-targeted senolytic delivery

Bone-targeted delivery systems may improve the therapeutic index of senolytics by increasing drug accumulation in skeletal tissue and reducing systemic exposure. A bone-targeted delivery strategy for senolytics was shown to eliminate senescent cells and increase bone formation in senile osteoporosis models (76). Such approaches are highly relevant because osteoporosis is a chronic condition requiring long-term safety. Nanocarriers, bisphosphonate-conjugated delivery systems, and bone-affinity peptides may help localize senolytic activity to bone marrow or mineralized tissue. Nonetheless, these systems remain preclinical and require detailed evaluation of biodistribution, immune effects, pharmacokinetics, and long-term toxicity.

### 7.6 Combination strategies in preclinical models

Senolytics are unlikely to replace established osteoporosis therapies in the near term. More plausible strategies include combining senolytics with antiresorptive agents, anabolic therapies, exercise, nutritional optimization, or anti-inflammatory interventions. For example, senolytics may improve the bone marrow microenvironment, while anabolic agents stimulate bone formation and antiresorptives suppress osteoclast activity. However, combination therapy requires careful evaluation to avoid overlapping toxicity, impaired remodeling, or excessive suppression of physiological repair.

## 8 Clinical evidence and translational status

Clinical translation of senolytics for osteoporosis remains at an early stage. Early human studies suggest that intermittent dasatinib plus quercetin can reduce senescent-cell markers in selected disease contexts (77). More directly relevant to osteoporosis, a Phase 2 randomized controlled trial evaluated intermittent D+Q in postmenopausal women and found that D+Q did not significantly reduce bone resorption in the overall cohort. However, exploratory analyses suggested that women with higher levels of a candidate biomarker of cellular senescence burden, defined by higher T-cell p16^INK4a^ mRNA levels in peripheral blood, may have greater skeletal responses, including increased bone formation markers, reduced bone resorption markers, and improved radius bone mineral density (78). It is important to note that this measurement reflects peripheral T-cell p16^INK4a^ expression as a surrogate marker rather than directly quantifying senescent-cell burden in bone marrow or demonstrating selective clearance of senescent T cells or other bone-marrow immune-cell subsets. These findings support biomarker-driven patient stratification but do not yet justify routine clinical use of D+Q for osteoporosis.

Overall, current clinical evidence should be interpreted as proof-of-concept rather than therapeutic confirmation. There is currently insufficient evidence to claim that any senolytic has established efficacy as an osteoporosis therapy in humans. Future trials should include adequate sample size, stratification by senescent-cell burden, standardized skeletal endpoints, bone turnover markers, high-resolution imaging, fall and fracture outcomes, and long-term safety monitoring. Having reviewed the preclinical models and early human trials, **Table 4** consolidates the empirical evidence for major senolytic and senescence-modulating strategies—including D+Q, fisetin, and bone-targeted platforms—while highlighting the preliminary nature of clinical data and the emergent role of biomarker-guided patient stratification.

**Table 4:** Summary of preclinical and clinical evidence.

Intervention	Model/study population	Main findings/effects	Evidence level and remarks	References
Dasatinib + Quercetin (D+Q)	Aged, postmenopausal, and radiotherapy-induced bone loss mouse models; Phase 2 trial in postmenopausal women	Preclinically: Reduced senescence markers, improved bone mass, microarchitecture, and regeneration; decreased marrow adiposity. Clinically: Phase 2 trial did not significantly reduce overall bone resorption, but exploratory subgroup with higher levels of a candidate biomarker of cellular senescence burden (higher T-cell p16INK4a mRNA in peripheral blood) showed potential benefits.	Robust preclinical evidence; clinical data remain preliminary and hypothesis-generating. Does not demonstrate lineage-specific clearance of senescent immune cells. The p16INK4a measurement is a surrogate marker rather than a direct quantification of senescent-cell burden in bone marrow.	(54, 60, 61, 70, 76–78)
Fisetin	Aging models and experimental osteoporosis models	Reduced senescence markers, inhibited osteoclast activity, improved bone microarchitecture, modulated RANKL/OPG signaling.	Promising preclinical data; mechanism (true senolysis vs. anti-inflammatory/antioxidant effects) requires further clarification.	(66, 74)
Astragaloside IV	D-galactose-induced bone loss mouse model	Attenuated macrophage senescence and bone loss via the STING/NF-κB pathway.	Primarily senescence-modulating rather than a classical senolytic; should be considered a senomorphic candidate unless selective elimination of senescent cells is demonstrated.	(32)
Navitoclax	Aged mouse models	Can eliminate some senescent cell populations; however, caused trabecular bone loss and impaired osteoprogenitor function in some studies.	Serves as a critical cautionary tale: senescent-cell clearance is not uniformly beneficial in the skeleton. Efficacy is context-dependent; toxicity (thrombocytopenia) and potential adverse skeletal effects limit its use in osteoporosis. The BCL-xL mechanism requires further investigation.	(64, 67, 75)
Bone-targeted delivery systems	Senile osteoporosis mouse models	Delivered senolytics specifically to bone, increased bone formation, and reduced systemic exposure.	Preclinical proof of concept; may improve therapeutic index but requires further evaluation of biodistribution, pharmacokinetics, and long-term toxicity.	(76)

This table consolidates empirical evidence from preclinical animal models (including aging, postmenopausal, and radiotherapy-associated bone loss models) and early-phase clinical trials, demonstrating the therapeutic potential of senolytic and senescence-modulating strategies—including dasatinib plus quercetin, fisetin, Astragaloside IV, navitoclax, and bone-targeted delivery platforms—in reducing senescent-cell burden and improving skeletal parameters, while also highlighting that clinical evidence remains preliminary, that patient subgroups with higher senescent burden may show more pronounced skeletal responses, and that senolytic efficacy cannot be assumed based solely on the ability to reduce global senescence markers. Bold text indicates critical caveats and cautionary findings.

D+Q, dasatinib plus quercetin; STING, stimulator of interferon genes; NF-κB, nuclear factor-κB; RANKL, receptor activator of nuclear factor-κB ligand; OPG, osteoprotegerin; BMD, bone mineral density; SASP, senescence-associated secretory phenotype.

## 9 Challenges and future directions

**Figure 4:**
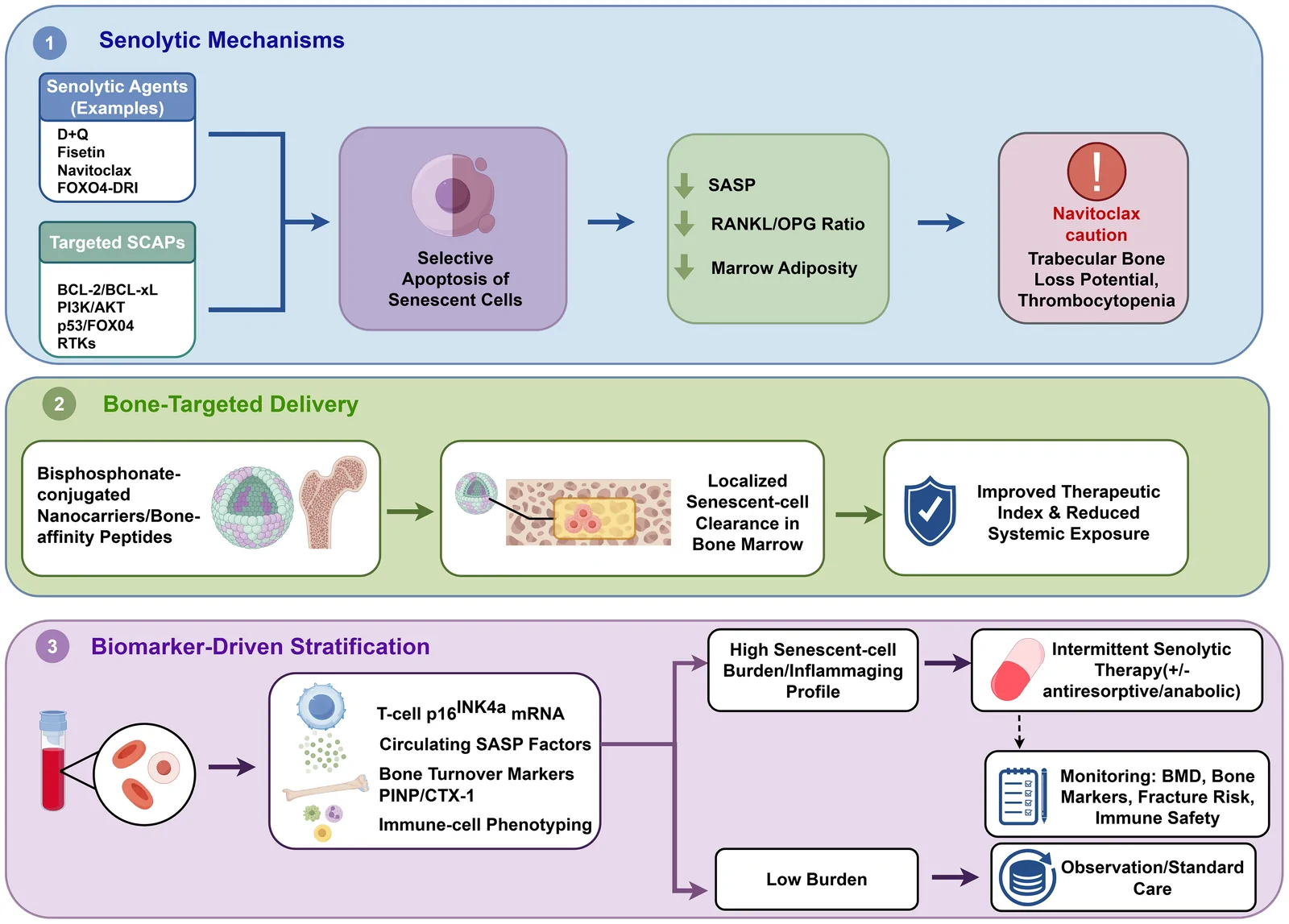
Senolytic mechanisms, bone-targeted delivery, and biomarker-driven patient stratification for osteoporosis therapy. Senolytics (e.g., dasatinib plus quercetin [D+Q], fisetin) exploit senescent-cell anti-apoptotic pathways (SCAPs), including B-cell lymphoma 2 (BCL-2) family proteins, phosphoinositide 3-kinase (PI3K)/AKT signaling, and the forkhead box O4 (FOXO4)-p53 interaction, to selectively eliminate senescent cells. Bone-targeted delivery platforms, such as bisphosphonate-conjugated systems, can increase skeletal drug concentration and reduce systemic exposure. Not all senolytics are uniformly beneficial; navitoclax may cause trabecular bone loss and thrombocytopenia. Given the heterogeneity of osteoporosis, biomarker-driven patient stratification using T-cell p16^INK4a^ mRNA, circulating SASP factors, and bone turnover markers is essential to identify individuals with high senescent-cell burden who are most likely to benefit from intermittent senolytic therapy, either alone or in combination with antiresorptive or anabolic agents. D+Q, dasatinib plus quercetin; SCAPs, senescent-cell anti-apoptotic pathways; BCL-2, B-cell lymphoma 2; PI3K, phosphoinositide 3-kinase; FOXO4, forkhead box O4. Figure drawn by www.figdraw.com.

The central challenges and proposed strategies—spanning the exploitation of senescent-cell anti-apoptotic pathways, bone-targeted delivery platforms, and biomarker-driven patient stratification—are schematically presented in **Figure 4**.

### 9.1 Defining senescent and senescence-like immune cells in bone

A major challenge is the lack of universally accepted criteria for identifying senescent or senescence-like immune cells in the bone marrow. Surface markers such as CD57, KLRG1, and CD28 loss are useful for defining late-differentiated T-cell states but are not sufficient to prove cellular senescence. Similarly, macrophage markers such as F4/80, CD11b, CD86, inducible nitric oxide synthase, arginase-1, and CD206 reflect lineage identity or polarization rather than senescence itself. Future studies should combine surface phenotyping with p16^INK4a^, p21^CIP1^, DNA damage markers, telomere status, senescence-associated β-galactosidase activity, mitochondrial dysfunction, apoptosis resistance, and SASP profiling.

As highlighted in Section 3.1 and applied throughout Sections 3.3-3.6, a major priority is the development of a more precise, multi-tiered classification system that distinguishes between classically senescent immune cells (requiring multi-marker validation), late-differentiated T cells, aged/dysfunctional innate cells, and pro-inflammatory/age-associated cells. Such a framework would enable more accurate interpretation of experimental findings and more targeted therapeutic development.

### 9.2 Achieving tissue selectivity and ensuring immune safety

The lack of lineage-selectivity is not just a mechanistic inconvenience; it is a primary safety concern (**Figure 4**). Because senescent cells and immune cells are both heterogeneous populations distributed across multiple tissues, systemic senolysis may have unintended and potentially deleterious consequences. Excessive or inappropriate depletion of senescent or senescence-like immune cells, which are integral components of the host defense system, could theoretically impair immune surveillance, increase susceptibility to infections, or disrupt normal tissue repair processes. This concern is particularly acute in the elderly osteoporotic population, in whom immune competence is already compromised by immunosenescence and who may face additional challenges from polypharmacy and comorbidities. Furthermore, as discussed in Section 6.5, senescent cells fulfill beneficial physiological roles in wound healing, tissue repair, and tumor suppression; their indiscriminate elimination could therefore compromise these processes. The navitoclax experience (Section 7.4) serves as an instructive example, demonstrating that senescent-cell clearance can be detrimental even within the bone microenvironment itself, depending on the cellular targets and context. Therefore, future therapeutic strategies must prioritize approaches that enhance selectivity for the desired senescent-cell populations within the bone marrow while sparing senescent cells in other tissues that contribute to normal physiology.

Tissue-selective delivery, local activation systems, antibody-drug conjugates, immune-cell-subset targeting, and bone-affinity carriers may improve specificity. Bone-targeted delivery systems, such as bisphosphonate-conjugated nanoparticles or bone-affinity peptides, have shown preclinical promise in concentrating senolytic agents within the skeletal compartment and reducing systemic exposure (76). However, these systems remain at an early stage of development and require detailed evaluation of biodistribution, off-target accumulation, pharmacokinetics, and long-term toxicity. Immune-cell-subset-specific targeting, through antibodies or ligands that recognize surface markers unique to senescence-associated T cells, macrophages, or other populations, represents a more distant but conceptually attractive goal. The feasibility of such approaches will depend on the identification of reliable, lineage-specific senescence-associated surface antigens, a task that will benefit from emerging single-cell and spatial omics technologies (Section 9.4). Long-term studies should monitor infection risk, malignancy risk, hematologic toxicity, wound healing, and immune competence as key safety endpoints. Ultimately, the successful translation of senolytic therapy for osteoporosis will require a careful balance between efficacy-reducing the senescence-associated inflammatory burden in bone-and safety-preserving the protective and reparative functions of senescent cells elsewhere in the body.

### 9.3 Biomarker-driven patient stratification

Clinical trials should not treat osteoporosis as a biologically uniform disease. Senolytics may benefit patients with high senescent-cell burden, pronounced inflammaging, elevated SASP factors, or specific immune-aging signatures. Candidate biomarkers include T-cell p16^INK4a^ expression, circulating SASP factors, inflammatory cytokine panels, bone turnover markers such as procollagen type I N-terminal propeptide and C-terminal telopeptide of type I collagen, immune-cell phenotyping, epigenetic aging measures, and imaging-based bone quality assessment. Biomarker-guided inclusion criteria may increase the likelihood of detecting therapeutic benefit.

### 9.4 Advanced technologies for mechanistic discovery

Single-cell RNA sequencing, single-cell ATAC sequencing, spatial transcriptomics, proteomics, metabolomics, and lineage tracing can help identify senescent immune-cell subsets within the bone marrow. These approaches may reveal whether specific T-cell, macrophage, neutrophil, or B-cell populations with senescence-associated features drive osteoporotic bone loss. Integrating multi-omics with functional assays and animal models will be essential for moving from association to causality.

### 9.5 Toward precision senolytic therapy for osteoporosis

The future of senolytic therapy in osteoporosis will likely depend on precision medicine. Patients with high senescent-cell burden, inflammatory bone loss, or specific immune-aging profiles may be more responsive than unselected populations. Drug selection may also need to be tailored according to the dominant senescent-cell type, skeletal compartment, comorbidity profile, and safety risk. Combining senolytics with bone-targeted delivery, established osteoporosis drugs, and lifestyle interventions may provide a more balanced therapeutic approach. Based on the preceding discussion of mechanistic uncertainties, safety concerns, and trial design issues, **Table 5** integrates the major translational challenges facing senolytic therapy in osteoporosis, alongside corresponding strategies and advanced technologies proposed to address them in future research.

**Table 5:** Translational challenges and future directions.

Challenge Area	Specific Challenges	Future Directions/Strategies	References
Defining senescent and senescence-associated immune cells in bone	Lack of universally accepted, specific criteria; surface markers such as CD57 or KLRG1 alone are insufficient to prove classical cellular senescence; true senescence vs. chronic activation/polarization remains difficult to distinguish.	Use multi-marker panels (p16^INK4a^, p21^CIP1^, γ-H2AX, telomere status, SA-β-gal activity, SASP profiling) combined with single-cell and spatial omics technologies for precise identification. Develop a multi-tiered classification system distinguishing classically senescent immune cells, late-differentiated T cells, aged/dysfunctional innate cells, and pro-inflammatory/age-associated cells.	(53–57)
Tissue selectivity and immune safety	Systemic senolysis may impair immune surveillance, tissue repair, wound healing, and tumor suppression; lack of lineage-selectivity is both a mechanistic and a safety concern.	Develop bone-targeted delivery systems (e.g., bisphosphonate-conjugated nanoparticles, bone-affinity peptides), antibody-drug conjugates, or subset-specific approaches to achieve selectivity; conduct long-term monitoring for infections, malignancies, and wound healing.	(67–69)
Patient stratification and biomarkers	Osteoporosis is heterogeneous; not all patients respond to senolytic therapy; unselected populations may show no overall benefit.	Identify predictive biomarkers (e.g., T-cell p16INK4a expression as a candidate biomarker of cellular senescence burden, circulating SASP-like factors, inflammatory cytokine panels, bone turnover markers) for precision medicine and biomarker-driven trial inclusion.	(77, 78)
Dosing regimens and long-term safety	Optimal intermittent vs. continuous dosing remains undefined; long-term safety in frail, multimorbid elderly populations is unknown.	Conduct well-designed randomized controlled trials with long-term follow-up, assessing fractures, infections, malignancies, and other hard clinical endpoints.	(67–69)
Mechanistic validation	Distinguish true senolysis (apoptosis of senescent cells) from senomorphic (anti-inflammatory, antioxidant, immunomodulatory) effects.	Include direct apoptosis assays (e.g., caspase-3/7 activation), lineage-specific senescent-cell depletion models, and rescue experiments in preclinical and translational studies.	(54, 55, 57–59)

This table consolidates the translational challenges and future directions discussed in the review, demonstrating the obstacles in defining senescent immune cells in bone marrow, achieving tissue selectivity for immune safety, developing biomarker-driven patient stratification, optimizing intermittent dosing schedules, and validating true senolysis versus senomorphic effects, and presents corresponding strategies to guide the future development of precision senolytic therapy for osteoporosis.

p16^INK4a^, cyclin-dependent kinase inhibitor 2A; p21^CIP1^, cyclin-dependent kinase inhibitor 1; γ-H2AX, phosphorylated histone H2AX; SA-β-gal, senescence-associated β-galactosidase; SASP, senescence-associated secretory phenotype.

Beyond identifying challenges, the conceptual framework presented in this review provides a structured roadmap for converting the central hypothesis—that senescent or senescence-like immune cells drive bone loss and that senolytics may counteract this process—into experimentally testable and clinically actionable evidence. Specifically, the tiered classification system introduced in Section 3.1 offers a standardized lexicon for categorizing immune cell states, enabling consistent interpretation across studies and facilitating cross-study comparisons. The proposed bidirectional feedback loop between senescent/senescence-like immune cells and secondary senescent stromal cells, while currently hypothetical, can be definitively tested through lineage-resolved experimental approaches. These include: (i) fate-mapping studies using inducible Cre-lox systems to track the origin, trajectory, and persistence of senescence-associated immune populations within the bone marrow niche over the course of aging and osteoporosis; (ii) lineage-specific senescent-cell depletion using transgenic models (e.g., p16^INK4a^- or p21^CIP1^-driven suicide genes) restricted to defined immune lineages to causally establish whether eliminating specific subsets—rather than global senescent-cell clearance—is sufficient to preserve bone mass; (iii) single-cell and spatial multi-omics (scRNA-seq, scATAC-seq, spatial transcriptomics) to deconvolute the cellular heterogeneity of the senescent-inflammatory niche, identify lineage-specific senescence-associated surface antigens, and map cell-cell interactions that sustain the self-amplifying inflammatory loop; and (iv) adoptive transfer and conditioned media experiments to dissect the paracrine versus autocrine contributions of SASP or SASP-like factors to secondary senescence propagation. Execution of these experiments would provide the causal evidence needed to transition the current hypothesis from correlative association to mechanistic understanding, thereby establishing a rational foundation for patient stratification and the design of lineage-targeted senolytic strategies in future clinical trials.

## 10 Conclusion

Immunosenescence and inflammaging are increasingly recognized as important contributors to age-related bone loss. Senescent or senescence-like immune cells may disrupt bone homeostasis by producing SASP factors that promote osteoclastogenesis, impair osteoblast function, induce stromal-cell senescence, and sustain inflammatory remodeling of the bone marrow niche. These mechanisms provide a compelling rationale for targeting senescent cells in osteoporosis; however, this represents a compelling hypothesis that requires rigorous testing, rather than an established mechanism.

Preclinical studies support the therapeutic potential of senolytic and senotherapeutic strategies, particularly D+Q, fisetin, senescence-modulating natural compounds, and bone-targeted delivery systems. However, the evidence remains more mature for global senescent-cell targeting than for selective elimination of senescent or senescence-like immune-cell subsets. Clinical evidence is still preliminary, and current data do not support routine clinical use of senolytics for osteoporosis outside clinical trials.

While the preceding discussion emphasizes the provisional nature of current evidence, it is equally important to recognize what this review affirmatively establishes. First, the tiered classification framework provides an operational ontology for characterizing immune cell states in the aging bone marrow, moving beyond the imprecise and often misleading umbrella term ‘senescent immune cells’ toward a nuanced categorization that distinguishes classically senescent cells, late-differentiated T cells, aged/dysfunctional innate cells, and pro-inflammatory/age-associated cells. This classification enables more precise experimental design, data interpretation, and cross-study comparison. Second, the synthesis of the SASP or SASP-like factor network and its context-dependent effects on bone cells establishes a mechanistic framework for understanding how immune-derived mediators—whether from truly senescent or merely dysfunctional immune populations—may disrupt the RANKL/OPG balance, promote osteoclastogenesis, and impair osteoblast function. Third, the articulation of the bidirectional feedback loop hypothesis provides a falsifiable model of how local inflammation and cellular senescence may become self-sustaining within the bone marrow microenvironment. Fourth, the critical evaluation of current senolytic evidence offers a sobering but essential benchmark: global senescence markers are insufficient to infer immune-cell-specific clearance, and lineage selectivity remains the paramount translational hurdle. Collectively, these contributions provide a conceptual scaffold that can guide hypothesis-driven research and accelerate the transition from descriptive observation to mechanistic understanding and, ultimately, to rationally designed clinical trials.

Future progress will require precise definition of senescent and senescence-like immune-cell populations, improved tissue-selective delivery, robust biomarkers for patient stratification, careful immune safety assessment, and well-designed randomized trials with clinically meaningful skeletal endpoints. If these challenges can be addressed, senolytics may become a valuable adjunctive strategy for treating osteoporosis by targeting the aging biology that underlies bone remodeling failure. With the implementation of the lineage-resolved experimental strategies outlined above, the translational barriers we have identified—while substantial—are eminently surmountable, and the path toward precision senolytic therapy for osteoporosis is both clearly charted and scientifically achievable.
